# Implantation of ascyrus medical dissection stent, our first-hand experience

**DOI:** 10.1186/s13019-023-02377-0

**Published:** 2023-11-22

**Authors:** Mouhammad Kanj, Benoit Cosset, Alexandre Oliny, Fadi Farhat

**Affiliations:** 1 Department of Cardiothoracic Surgery, Faculty of Medical Science, Lebanese University, Beirut, Lebanon; 2 Department of Cardiovascular Surgery, Louis Pradel Hospital, Lyon, France; 3 Department of Cardiovascular Surgery, Infirmerie Protestante, 1-3 Chemin du Penthod, 69300 Caluire et Cuire, Lyon, France

**Keywords:** Acute aortic dissection, AMDS, Remodeling, Radial Force

## Abstract

**Background:**

Acute type A aortic dissection is associated with high perioperative morbidity and mortality. Ascyrus Medical Dissection Stent (Cryolife, Kennesaw, USA) is a novel uncovered hybrid stent graft developed to be used as an adjunct to standard surgical approach to promote true lumen expansion and enhance aortic remodeling.

**Methods:**

From March 2021 to March 2022, four consecutive patients presented with acute Debakey type I aortic dissection and underwent emergent surgical repair with Tirone David procedure and implantation of Ascyrus Medical Dissection Stent. We reviewed patient’s files retrospectively and described the perioperative outcomes.

**Results:**

All four device implantations were successful. Overall 30-day mortality was 0%. Malperfusion that was present in two patients pre-operatively improved after Ascyrus Medical Dissection Stent implantation. No aortic reinterventions were needed. No aortic injury related to the device was noted. Favourable changes in aortic true lumen and false lumen dimensions were found in most of our patients but the stent was compressed at the isthmus in one patient.

**Conclusion:**

Ascyrus Medical Dissection Stent is a reliable and secure device. However, its benefits remain unclear when it comes to a positive remodeling and seems less likelihood comparable to a frozen elephant trunk. The main reason seems to be an insufficient radial force of the stent.

## Introduction

Acute Debakey type I aortic dissection is a debilitating disease that may be complicated by organ malperfusion in 30% to 40% and carries high mortality rate [1–3]. The standard surgical repair by hemiarch reconstruction successfully manage the primary entry tear by resection; however, the distal false lumen remains depressurized and the true lumen is incompletely expanded. This may result in ineffective treatment of malperfusion. The Ascyrus Medical Dissection Stent (AMDS; Cryolife, Kennesaw, USA) is a new device developed to be used in adjunct to current surgical aortic dissection repair and is designed to improve short-term malperfusion syndrome and long-term aneurysmal evolution. Bozso et al. have shown on the DARTS trial the security and feasibility of the AMDS, and the capacity of this device to improve aortic remodeling [4, 5]. Mehdiani et al. also showed that AMDS can be safely performed in patients who need partial replacement of the aortic arch beyond zone 0 [6]. Herein, we report the first French implantation of the AMDS.

## Patients and methods

### Patients characteristics and data collection

During a 1-year period (March 2021 through March 2022), four consecutive patients received the AMDS hybrid prosthesis at the decision of the same attending surgeon for type I Debakey aortic dissection (Table 1). During the study period, 35 patients were managed for type A aortic dissection. All of the patients had an open distal anastomosis (hemi or total arch replacement) regarding our institution policy. Indications for AMDS implantation were most likely dynamic malperfusion syndromes as in alternative to a frozen elephant trunk. All procedures were done in a single institution in France (Louis Pradel University hospital, Lyon). Demographic data, comorbidities, operative procedures and post-operative variables were analysed retrospectively. Baseline characteristics of the study population are summarized in Table 2.

**Table 1:** AMDS configuration according to the pre-op CT scan measurements of the aortia diameter (adventitia to adventitia) at different zones

(Zone 1)/proximal aortic diameter: between the innominate and left common carotid artery (mm)	(Zone 4)/distal aortic diameter: at the level of the tracheal bifurcation (mm)	AMDS configuration	Length of device at the specified minimum diameter* (mm)	Length of device at the specified maximum diameter* (mm)
20–35	20–24	AMDS 40–30 (tapered)	208	155
20–35	35–35	AMDS 40 (straight)	210	170
36–45	27–35	AMDS 55–40 (tapered)	231	195
36–45	36–45	AMDS 55 (straight)	225	190

*Maximum length of the stent when it is expanded to accommodate the smallest aortic diameter

**Table 2:** Baseline characteristics of the study population

Baseline characteristics	Value
Age (years)	61 (51–69)
Male gender (%)	75 (3/4)
Malperfusion (%)	50 (2/4)
Preoperative stroke (%)	25 (1/4)
Hypertension (%)	100 (4/4)
COPD (%)	0
Reoperation	0

#### Case 1

The first patient is a 65 years old man with a medical history of HTN presenting for severe, sudden onset migratory chest pain that started few hours before presentation. On physical examination, the patient had high blood pressure reaching 180/90 mmHg. He was fully conscious without neurological deficit and the rest of physical examination was unremarkable. The EKG showed sinus rhythm with no signs of ischemia. A computed tomography (CT) angiography revealed a type A aortic dissection starting in the aortic root and extending through the aortic bifurcation until the origin of the left common iliac artery. There was also a dynamic stenosis at the origin of the hepatic artery but liver enzymes were in normal range. Other visceral arteries were normal and there was no evidence of malperfusion syndrome. The Supra-aortic trunks were normal. IV antihypertensive drugs were started and the patient was referred for emergent surgical treatment. Intraoperatively, the primary entry tear was found in the ascending aorta immediately distal to the origin of the Right Coronary Ostia (RCO). The aortic root was aneurysmal and involved in the dissection but the aortic valve was normal. Upon these findings, we decided to do a valve sparing repair. Tirone David procedure was done using a GELWEAVE™ Valsalva Dacron tube 28 mm (Terumo Aortic). Exploration of the arch showed no entry tears. An AMDS 55DE was implanted in the aortic arch and the descending thoracic aorta. Cardiopulmonary bypass time was 105 min, cerebral perfusion time was 15 min and cross clamping time was 87 min. On post-operative day (POD) 1, the False Lumen (FL) decreased in size but remained patent. The patient remained 4 days in the intensive care unit and was discharged home on POD 13 without major complications. On POD 30 the FL was partially thrombosed, it remained stable in size at the level of aortic arch and the Descending Thoracic Aorta (DTA), but increased in size at the level of the isthmus. The visceral arteries aroused from the true lumen with the exception of the inferior mesenteric artery (IMA). A new entry tear was identified in the abdominal aorta far below the distal AMDS tip.

#### Case 2

The second patient is a 59 years old man known to have HTN presenting to the emergency department for severe chest pain that appeared during wood chopping few hours prior to presentation. The patient was conscious and the physical examination was unremarkable. He had symmetric blood pressure. Blood tests showed Acute Kidney Injury (AKI) with decreased Glomerular Filtration Rate (GFR) to 37 ml/kg/1.73 m^2^. EKG showed diffuse ST depression and troponin test was positive. He was first misdiagnosed as having primary myocardial infarction and loaded with ticagrelor and low molecular weight heparin (LMWH). Pain was resistant to nitrates. A CT angiography was subsequently done and revealed acute type A aortic dissection starting in the aortic root and extending to the external iliac arteries. There was dynamic intestinal hypoperfusion due to compression of the true lumen by the false lumen from the level of diaphragmatic hiatus to the level of renal arteries. Dissection also involved the Brachiocephalic Artery (BCA) proximally. The patient was referred for emergent surgery. The pre-operative transesophageal echocardiography (TEE) showed an intimal flap at the level of the STJ with severe Aortic Insufficiency (AI) and normal biventricular function. Upon exploration, the entry tear was found in the ascending aorta just distal to the RCO and the dissection was totally circumferential in the aortic root. The aortic valve was tricuspid and without lesions. In these circumstances, it was possible to do a conservative intervention. Tirone David procedure was done using a GELWEAVE™ Valsalva Dacron tube 30 mm (Terumo Aortic). Exploration of the arch showed no entry tears. The distal aorta was managed with implantation of an AMDS 55DE in the arch and descending thoracic aorta. This patient needed repair of both coronary ostia before being re-implanted in the Dacron tube. In fact, the dissection reached both coronary ostiae circumferentially and repair was done with multiple sutures to reattach the intima to the coronary artery wall. Weaning of CPB was done after 123 min on inotropes and vasopressors. Cerebral perfusion time was 14 min and aortic cross-clamping time was 93 min. The immediate post-operative course was marked by continuous bleeding in the first few hours that necessitated administration of blood transfusions along with Tranexamic Acid without the need of re-exploratory sternotomy. There was also right ventricular dysfunction and AKI with the need of two dialysis sessions. Furthermore, the patient developed right brachial plexus palsy with partial recovery over time. After 10 days in the ICU, the patient was transferred to the regular floor where he continued to evolve in good conditions. He was discharged home on the POD 20 with a LVEF of 55% and a GRF of 38 ml/kg/1.73 m^2^. The follow-up post-operative CT angiography showed FL obliteration at the level of the arch and partial thrombosis at the TDA. However, the stent was collapsed at the isthmus where the FL remained patent without obvious entry tear. The celiac trunk, the SMA and the right renal artery aroused from the true lumen and were all permeable. The IMA aroused straddling the true and the false lumen, it was permeable but thin distally. No distal anastomosis induced new entry tears were observed.

#### Case 3

The third patient is a 51 years old lady with Marfanoid body habitus and a past medical history of HTN presenting to the emergency department for severe chest pain starting in the morning. Her physical examination was normal beside asymmetric blood pressure in the upper extremities. A CT angiography was done and showed type A aortic dissection extending to the BCA at its proximal third, the left Subclavian Artery, the celiac trunk and the SMA, with dynamic hypoperfusion. The patient was referred for surgical treatment. Pre-operative TEE revealed normal biventricular function, a dilated ascending aorta of 53 mm maximal diameter and an intimal flap at the level of the STJ extending through the aortic valve and creating a severe AI. Upon exploration, the aorta was found aneurysmal at its root and its ascending part. The primary tear was found just distal to the RCO. It extends in the non-coronary sinus to end distal to the left main coronary artery. The aortic valve was tricuspid without lesions. Thus, a Tirone David repair was performed using a GELWEAVE™ Valsalva Dacron tube 28 mm (Terumo Aortic). After excluding the entry tears in the aortic arch, an AMDS 55DE was implanted to manage the distal aorta. The cross-clamping time, cerebral perfusion time and cardiopulmonary bypass time were 84, 18 and 108 min respectively. The patient was transferred to the ICU on moderate doses of vasopressors but with a high lactate level 8.7 mmol/L with respect to the pre-operative level that was 1.7 mmol/L (normal level < 2 mmol/L). In the immediate post-operative hours, high volume of fluid administration enabled weaning from vasopressors and improvement of the lactate level slowly. The patient was extubated on the POD 1. Hemoglobin level was decreasing without obvious cause and the patient had abdominal pain. A CT scanner of chest, abdomen and pelvis was done on the POD 2 and showed an intraperitoneal hematoma without evidence of active bleeding. The false lumen at the level of the SMA was stable in size. There was a global colectasia, and abundant intraperitoneal effusion associated with diffuse submucosal edema of the colon with slightly altered enhancement, particularly of the left colic angle and the descending colon. Exploratory laparotomy was done by the general surgery team. There was no evidence of mesenteric ischemia. More than one liter of blood was drained from a hematoma at the level of the gastro-colic ligament without any evidence of active hemorrhage from the abdominal viscera. Bowel walls were normal without any suspected lesions. Finally, abundant lavage of the abdominal cavity was done before closure of the laparotomy. The patient remained ten days in the ICU and was discharged home on the POD 18 with a preserved left ventricular function and no residual AI. The follow-up CT angiography on the POD 1 and POD 30 showed FL obliteration at the arch and partial thrombosis at the isthmus and TDA without evidence of any re-entry tear.

#### Case 4

The fourth patient is a 69 years old man with a past medical history of HTN who presented to the emergency department for confusion and agitation. CT Angiography scanner established the diagnosis of type A aortic dissection starting in the ascending aorta and extending to the common iliac arteries without involvement of the supra-aortic trunks nor the mesenteric arteries. The patient was then referred for surgical repair. The entry tear was located in the ascending aorta and the dissection involved the aortic root without reaching the aortic valve nor the coronary ostia. The aortic valve was free of lesions. A valve sparing repair was possible in this situation. We did a Tirone David procedure along with implantation of an AMDS in the aortic arch and the descending thoracic aorta after excluding the presence of any intimal tear in the aortic arch. The cross-clamping time, cerebral perfusion time and cardiopulmonary bypass time were 79, 15 and 116 min respectively. The patient was transferred to the ICU on low dose of norepinephrine that was weaned few hours later. He developed paroxysmal rapid atrial fibrillation with hemodynamic instability and received pharmacological and electrical cardioversion to restore sinus rhythm. When sedation was stopped, the patient developed severe agitation. MRI of the brain showed multiple cerebral small emboli. He also developed ventilator-associated pneumonia and acute renal insufficiency requiring hemodialysis. After ten days in the ICU, the patient regained full consciousness and was extubated. His renal function improved without the need for further hemodialysis. After 22 days in the ICU, he was transferred to the regular floor where he continued to evolve in favourable conditions. He was finally discharged home on POD 31 with preserved biventricular function and no AI. The Follow-up CT angiography on the POD 1 and POD 30 showed FL obliteration at the aortic arch and partial thrombosis at the isthmus and TDA. The abdominal visceral arteries arised from the true lumen and were all patent. There was no evidence of additional new entry sites.

### Device description

**Fig. 1:**
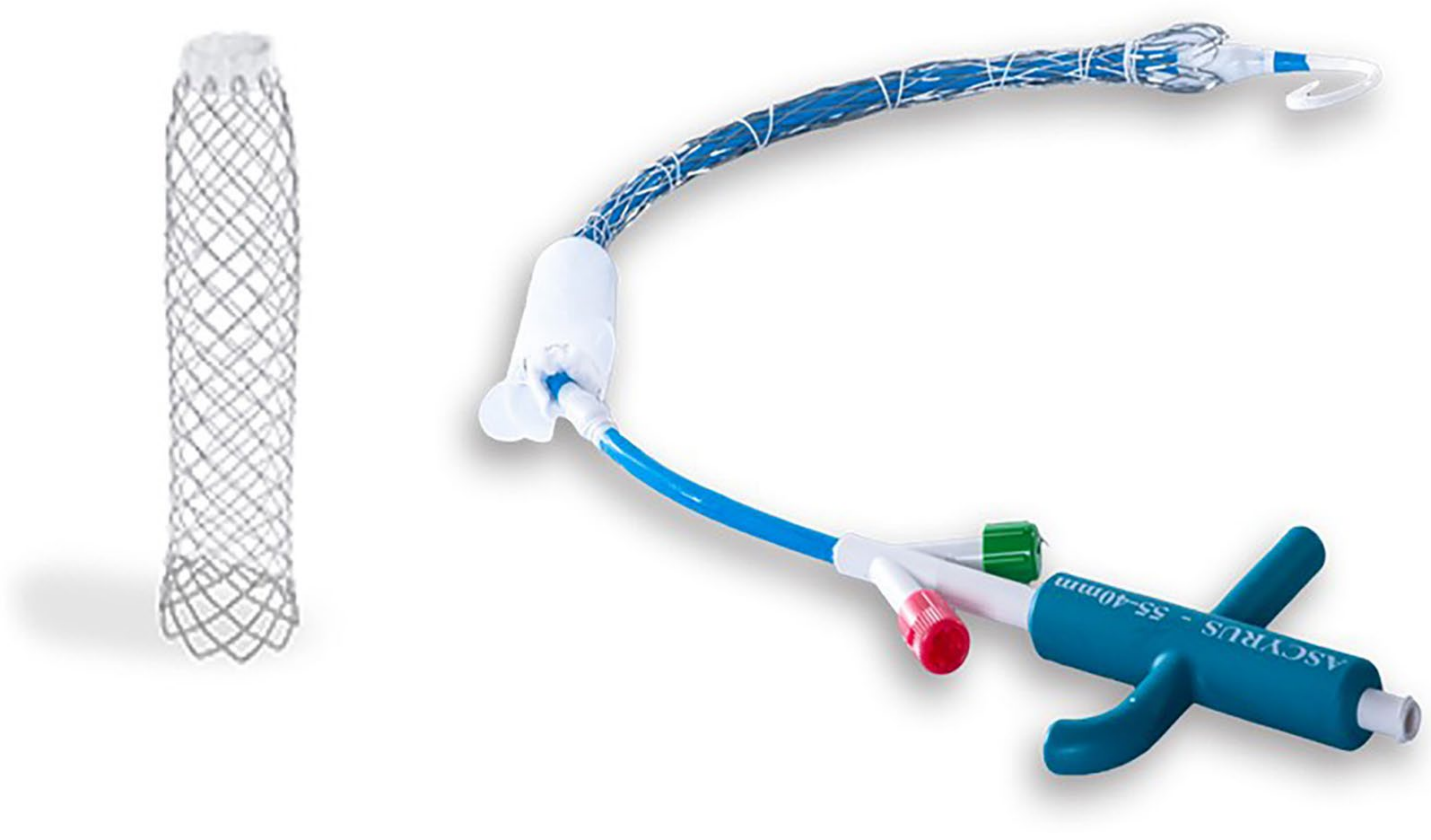
Fully Expanded Ascyrus Medical Dissection Stent (AMDS) and contained within its delivery system

The Ascyrus Medical Dissection Stent (AMDS, Cryolife, Kennesaw, USA) is a new device developed to be used in adjunct to current surgical aortic dissection repair and is designed to improve short-term malperfusion syndrome and long-term aneurysmal evolution. This hybrid prosthesis is composed of proximal PTFE felt graft and a distal uncovered Nitinol wire braided stent with low outward force (Fig. 1). It is designed for antegrade implantation during hypothermic circulatory arrest into the aortic arch and the descending thoracic aorta. Four different sizes exist to accommodate a range of aortic diameters (Table 1). The required sizing is performed preoperatively using a multiplanar computed tomography (CT) scan with 2 aortic landmarks: in the aortic arch (zone 1) and the descending aorta at the tracheal bifurcation (zone 4). The AMDS is mounted on a delivery system that includes an internal shaft onto which the device is loaded, a protective sheath, a deployment mechanism, and a handle. Once the device is introduced into the aorta, the transparent protective sheath covering the PTFE ring is removed and discarded. The PTFE ring is sutured to the edge of the transected aorta ensuring that the PTFE does not obstruct the orifices of the branches of the aortic arch. Unscrewing the green cap releases the suture holding the stent in place, allowing the stent to expand.

### Operative technique

All patients underwent emergency surgery under cardiopulmonary bypass (CPB) initiated with arterial cannulation of the right axillary artery and venous cannulation of the right atrium. After lowering body temperature, the ascending aorta was cross-clamped and the prevention of myocardial ischemia was achieved through antegrade cold crystalloid cardioplegia followed by intermittent administration of selective coronary cardioplegia throughout the clamping time. Longitudinal aortotomy was performed, followed by resection of the aortic root while preserving 3 to 4 mm of aortic stump above the aortic valve annulus. Coronary ostial buttons were then prepared. Six subannular U-points were placed and anchored to a GELWEAVE™ Valsalva Dacron tube (Terumo, Aortic), 28 or 30 mm in diameter. Finally, the aortic valve commissures were attached within the tube.

At rectal temperature of 28 °C, and under cerebral monitoring by Near-infrared spectroscopy (NIRS), hypothermic circulatory arrest was started and the aorta was declamped. Cerebral perfusion was ensured by starting the flow of blood (7.5–10 ml/kg/min) into the right subclavian artery while clamping the origins of the Brachiocephalic artery and the Left Common Carotid artery. Exploration of the aortic arch demonstrated no entry tears in all patients. Transection of the aorta was then performed 1 cm proximal to the origin of the Innominate trunk. A 55 mm uncovered AMDS was then implanted over a guidewire in the true lumen of the aorta. Distal anastomosis between the aorta and the AMDS collar was done using a new Dacron tube reinforced by an external Teflon felt. CPB was then re-initiated, the aortic arch was purged, the Dacron tube was clamped and rewarming was commenced until reaching normothermia.

During the rewarming time, the aortic valve was re-implanted to the Valsalva Dacron tube using running 4.0 Polypropylene sutures. Re-implantation of the coronaries into their respective ostia then followed. In one patient, the dissection reached the coronary ostia and repair was done using multiple sutures to reattach the intima to the coronary artery wall. End-to-end anastomosis between the two Dacron tubes was done using Polypropylene 3.0 running sutures. After de-airing the cardiac cavities, the tube was declamped and CPB was weaned down.

### Statistical analysis

Statistical analysis and graphics were done using statistical softwares (IBM® SPSS® Statistics version 26.0, Armonk, NY: IBM Corp; GraphPad Prism® version 8.0, for Windows, GraphPad Software, San Diego, California USA, www.graphpad.com).

## Results

### Baseline characteristics

**Fig. 2:**
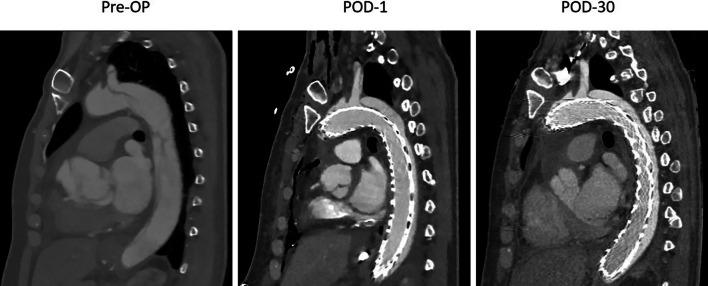
Computed tomography angioscanner showing the deployed AMDS in the true lumen at pre-op, POD1 and POD30

The baseline clinical characteristics are presented in Table 1. Mean age was 61(± 8) years old and 3 patients were male (75%). One patient had Marfanoid habitus. All patients had a previous medical history of hypertension and chronic kidney disease and no one was smoker. Mean BMI was 24.5(± 2) kg/m^2^ and mean Euroscore-II was 22(± 18) %. CT-Angiography was done pre-operatively and on post-operative day 1, day 30 for all patients (Fig. 2). Primary tear was identified in the ascending aorta in all patients. Two patients (50%) had evidence of dynamic intestinal malperfusion by compression of the true lumen. One of those patients had also evidence of coronary malperfusion and severe aortic insufficiency. Another patient had acute neurological deficit pre-operatively marked with aphasia and decreased consciousness.

### Operative data

A re-implantation technique (David V) was performed in all patients along with implantation of the AMDS. Surgical and perioperative data are presented in Table 3. The mean cross clamping time, cerebral perfusion time and cardiopulmonary bypass time were 85(± 6), 15(± 2) and 113(± 8) minutes respectively. Circulatory arrest was attempted at a body core temperature of 28°C.

**Table 3:** Procedural data

Characteristic	Value
Successful device deployment	100%
David V procedure	100%
Mean cardiopulmonary bypass time (min)	113 (± 8)
Mean cross clamping time (min)	85 (± 6)
Mean cerebral perfusion time (min)	15 (± 2)

### Postoperative outcomes

Mortality and serious adverse events are summarized in Table 4. One patient had smooth postoperative course without complications. The patient who had severe Aortic insufficiency with coronary and intestinal malperfusion developed right ventricular dysfunction and required hemodyalisis post-operatively. He also developed right brachial plexus palsy with partial recovery. He has no more intestinal malperfusion. The other patient with intestinal malperfusion developed hemorrhagic shock post-operatively with severe abdominal pain. An exploratory laparotomy revealed a huge hemoperitoneum that was drained. The patient remains having intestinal angina but with clinical improvement. The patient who had neurological deficit developed multiple small cerebral emboli due to atrial fibrillation. He also developed ventilator-associated pneumonia and acute renal insufficiency requiring hemodialysis. His consciousness improved with time. No aortic reinterventions were needed. No aortic injury related to the device was noted. Mortality rate was zero at the post-operative day 30. Patients were discharged from the hospital after an average of 21 days.

**Table 4:** Mortality and serious adverse events

Mortality and serious adverse events	Value
30-day mortality	0%
Neurological deficit	25% (1 patient)
Acute renal failure requiring hemodialysis	50% (2 patients)
Hemorrhagic shock	25% (1 patient)
Aortic injury associated with device implantation	0%
Device related reintervention	0%
Stent compression	25% (1 patient)

### Comparison of CT measurements

**Fig. 3:**
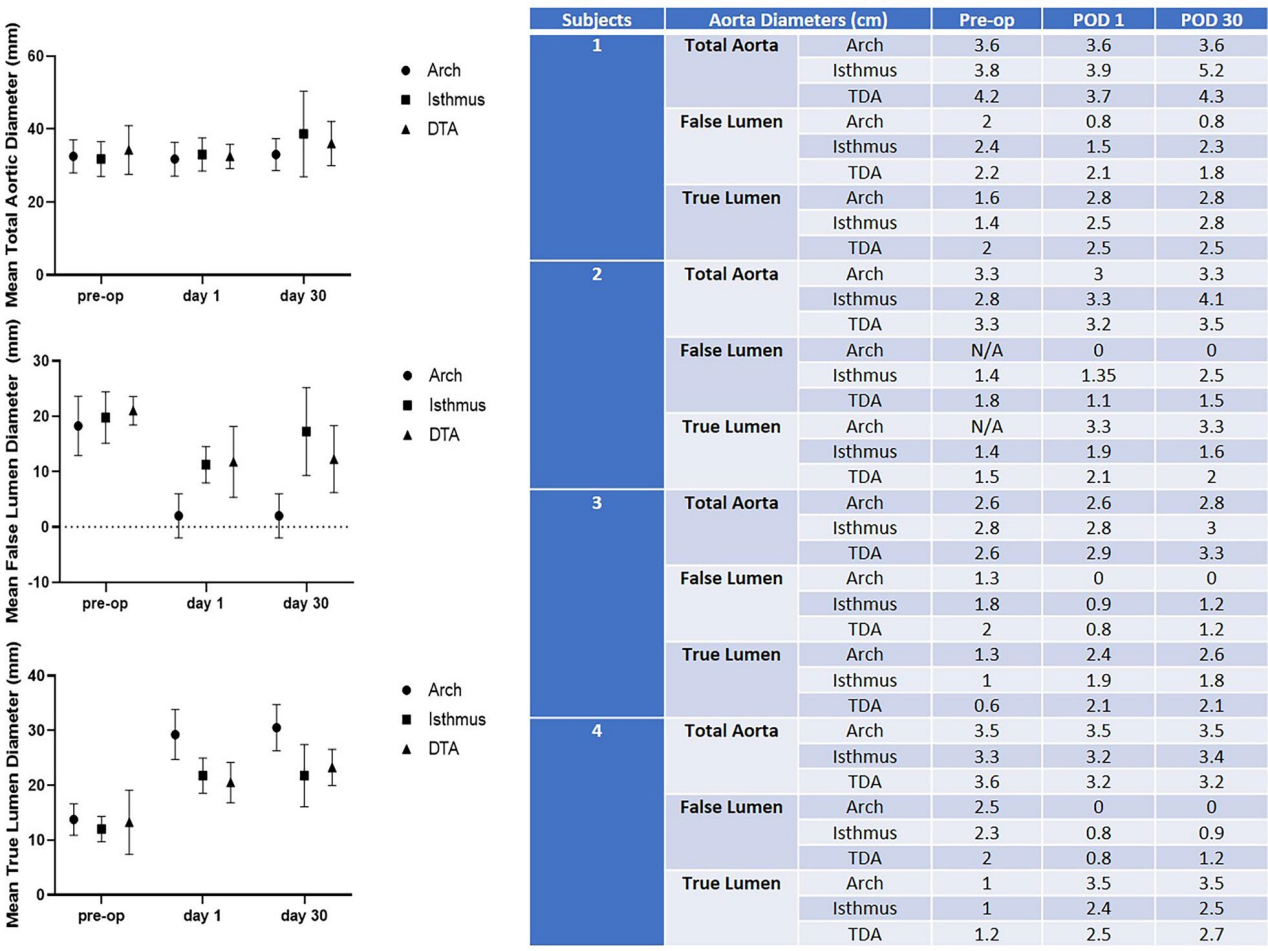
Comparison of the CT measurements of the different aortic diameters

Figure 3 shows comparison of CT measurements of the different aortic diameters.

**Fig. 4:**
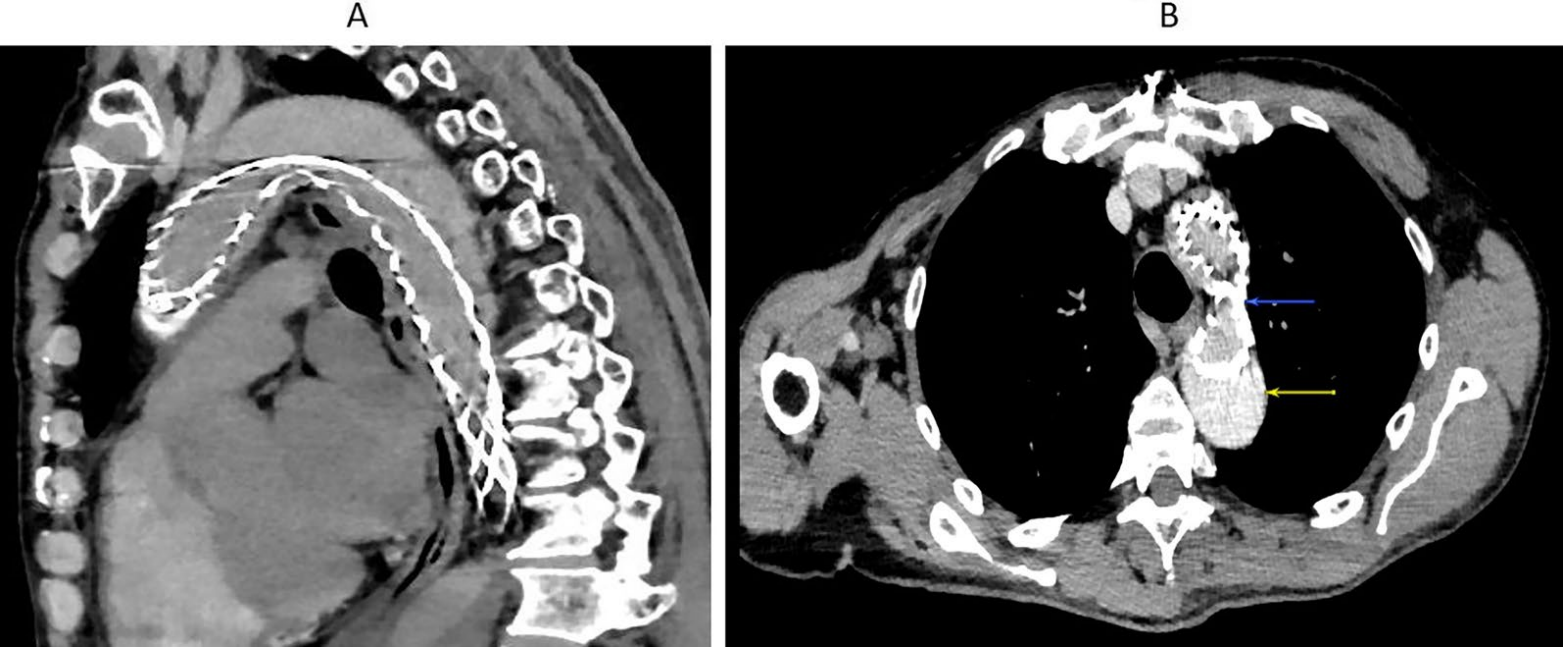
**A** Compression of the AMDS at the isthmus in one patient. **B** Yellow arrow: opacification of the false lumen. Blue arrow: stent compression

The mean total aortic diameter remained stable at the aortic arch and descending thoracic aorta in all patients. It increased at the isthmus in one patient (by 31%). The false lumen was obliterated at the aortic arch in three patients and decreased in size (by 60%) in the fourth one. At the level of the isthmus it increased in only one patient because of reperfusion. However, at the level of the descending thoracic aorta, the mean false lumen size decreased in all patients (by 43%). Concerning the mean true lumen size, it increased in all patients (by 91%) compared with baseline. However, the stent was compressed in one patient at the level of the isthmus (Fig. 4). No distal anastomosis re-entry tear was observed. A new entry tear was identified in one patient at the level of abdominal aorta far below the distal AMDS tip.

## Discussion

Acute Debakey type I aortic dissection has been preferably managed by hemiarch aortic reconstruction. This approach had a main disadvantage; it may leave entry tears in the proximal descending thoracic aorta leading to false lumen patency in majority of patients increasing the need for re-operation [7–10]. Total arch replacement with Frozen Elephant Trunk allows for distal extension of a stent graft implant into the true lumen of the descending aorta excluding re-entry tears in the arch and proximal descending thoracic aorta. In the setting of acute aortic dissection and deeply ill patients, this approach can increase the CPB and circulatory arrest time, and exposes patients to additional risks of paralysis, stroke and haemorrhage [11, 12]. It may be more useful in particular situations in the setting of aortic dissection like when there is arch aneurysm, or there is an entry tear within the arch, or when there is rupture to the proximal descending thoracic aorta associated with the dissection [13].

Between these two approaches, the AMDS represents a novel hybrid solution providing long thoracic coverage alleviating malperfusion and excluding entry tears without significantly increasing the complexity of surgery [4–6].

DARTS trial has shown a good rate of aortic remodeling. However, this was a composite criterion; positive remodeling was defined on evidence of false lumen obliteration, complete false lumen thrombosis and favourable changes in aortic dimensions [14, 15]. Our real-life experience shows that these results are more the consequence of lumen’s diameters correction than a complete false lumen thrombosis, which is possibly due to the primary entry tear exclusion.

There was no need for a redo surgery in our four patients, but we think that the presence of Nitinol in the arch jeopardizes a second procedure at this level.

In the two patients having intestinal malperfusion we noticed clinical improvement. One of them remains having intestinal angina due to dissection of the SMA. Favourable changes in aortic true lumen and false lumen dimensions were found in most of our patients but the AMDS was compressed at the isthmus in one of them. We did not have any mortality in our patients. This shows that AMDS is a reliable and secure device. However, its benefits remain unclear when it comes to a positive remodeling and seems less likelihood comparable to a frozen elephant trunk. The main reason seems to be an insufficient radial force of the AMDS, which tend to lengthen rather to expand. Many studies addressing the radial force of endovascular stents emphasized on the importance of understanding radial force when selecting a stent for every patient. Radial force of endovascular stents provides effective support for blood vessels, maintains adequate lumen patency, and secures fixation to artery wall [16]. It varies among stent designs, and differences depend on the type of stents, the site of deployment or layer characteristics of each stent [17, 18]. In vivo, endovascular stents would be affected by the vessel curvature, blood pressure, vascular smooth muscles characteristics and much more dynamic factors [17]. Surgeons should evaluate the possibility of stent deformities during and after surgery [19]. It is believed that treatment of dissection with endovascular stent requires fewer radial force compared with the treatment of aneurysm because too much radial force at distal ends may lead to stent graft induced new entry tears (SINE). The reported incidence of SINE may reach 25%, and its occurrence is typically delayed. It is usually discovered on routine imaging follow-up one to three years after endovascular treatment. In chronic aortic dissection, the dissected intimal flap thickens and becomes less mobile. Therefore, persistent radial force exerted by the distal compressed stent graft may lead to SINE over time. However, in acute aortic dissection, the intimal flap is still highly mobile. This will allow the distal end of the stent graft to fully expand maintaining the dissection flap to its previous anatomic position adjacent to the outer aortic wall [20]. However, the arch geometry for thoracic aorta requires larger radial forces to seal [21]. In its initial experience on AMDS stent, Montagner et al. concluded that the low radial force of AMDS stent is intended just to readapt the intima against the media and adventitia and it is the subsequent expansion of the TL that will drive the resolution of malperfusion [22]. Furthermore, they emphasized on the importance of low radial force of the AMDS stent which can unlikely damage the intima. They also reported three failures of device deployment, one of them being due to high turtosity of the aorta causing kinking and incomplete AMDS expansion [23]. In addition to the low radial force, the appearance of new entry tears could maintain patency of the FL and this will lead to more expansion of the FL at the expanse of the low radial force AMDS. Figure 4B shows opacification of the false lumen on control CT angiography without being able to identify the entry site. Another possible cause of stent compression could be the oversizing. In fact, in two of our four patients, the AMDS was oversized to overcome the high radial force of the aortic arch. In one of them the stent collapsed at the isthmus. Low radial force of the AMDS should not lead surgeons to oversize it. Indeed, an insufficiently expanded stent in the TL may collapse over time. So, oversizing should be avoided and we advise surgeons to commit to the sizes proposed by the manufacturer.

Even though we observed improvement of malperfusion, this may be attributed only to primary entry tear resection. Actually, the radial force of the AMDS is not adequate to guarantee distal expansion of the true lumen, especially in case of visceral malperfusion and this was seen in one of our patients. AMDS implantation should be avoided in patients with aortic calcifications or kinking to prevent incomplete stent expansion.

Another feature should be mentioned is that the AMDS is an uncovered stent. It has the advantage to maintain patency of the aorta by pressurizing the TL the without neither occluding any branch of the arch vessels nor adding additional risk for spinal cord injury. However, the main drawback in bare stents resides in their inability to exclude any entry tear in contrast to covered grafts that allow immediate closure of entry tears while maintaining patency of the parent vessel. In this condition, any entry tear not excluded by this bare stent will remain active. Indication for the AMDS implantation should be limited to type A aortic dissection with a primary tear in the root or the ascending aorta. The arch should be absolutely free from any tear. Patients with factors that could impede stent expansion such as aortic calcifications or kinking should be avoided.

## Limitations

The main limitations of this study remain in its small sample size, its retrospective design and the absence of long-term follow-up. Experimental and clinical studies with larger AMDS patient cohorts to clarify potential risks and to investigate on the potential benefits such as the positive aortic remodelling during follow-up are recommended.

## Conclusion

This initial study suggests that the AMDS is safe, feasible and reproducible adjunct to the standard surgical repair for acute Debakey type I aortic dissection without extending the procedure time. However, its benefits remain unclear when it comes to a positive remodeling.

## Data Availability

All data and images are available.
